# Association of serum creatinine/cystatin C ratio with insulin resistance and all-cause mortality: a national cohort analysis

**DOI:** 10.3389/fnut.2025.1619618

**Published:** 2025-08-29

**Authors:** Yutong Han, Jiahong Zhang, Qian Li, Bin Wan, Chunguang Xie

**Affiliations:** ^1^Chengdu University of Traditional Chinese Medicine, Chengdu, China; ^2^TCM Regulating Metabolic Diseases Key Laboratory of Sichuan Province, Hospital of Chengdu University of Traditional Chinese Medicine, Chengdu, China; ^3^Department of Endocrinology, Hospital of Chengdu University of Traditional Chinese Medicine, Chengdu, China

**Keywords:** cystatin C, insulin resistance, mortality, creatinine, biomarker

## Abstract

**Background:**

Serum creatinine/cystatin C (Cr/CysC), a biomarker for skeletal muscle mass, has not been well studied in relation to insulin resistance (IR). This study examined the associations between Cr/CysC, IR, and all-cause mortality.

**Methods:**

Data were sourced from the NHANES database and analyzed using logistic and linear regression to assess the association between Cr/CysC and IR, quantified by the triglyceride-to-high-density lipoprotein cholesterol (TG/HDL) ratio. Restricted cubic splines (RCS) were employed to identify non-linear associations, and Cox regression was leveraged to determine associations with all-cause mortality.

**Results:**

Higher Cr/CysC ratios were strongly associated with lower IR risk (OR = 0.48, 95% CI: 0.32–0.73, *p* = 0.001) and lower TG/HDL (*β* = −0.60, *p* = 0.001). RCS analysis indicated a non-linear relationship, with increased IR risk below a certain threshold (*p* < 0.05). Cox regression revealed a negative association between Cr/CysC and all-cause mortality in the overall population (HR = 0.47, 95% CI: 0.31–0.69, *p* < 0.001) and among non-IR individuals, but not among those with IR. Associations were stronger in middle-aged individuals, women, and non-hypertensive participants.

**Conclusion:**

Cr/CysC is inversely associated with IR and all-cause mortality, suggesting its potential as a low-cost marker for stratifying IR risk.

## Introduction

Insulin resistance (IR) is a pathological condition defined by disrupted glucose uptake and usage in the liver, skeletal muscle, adipose tissue, and other insulin-target tissues (1, 2). IR is strongly associated with various metabolic disorders, such as metabolic syndrome (3, 4), polycystic ovary syndrome (5, 6), non-alcoholic fatty liver disease (7, 8), and cardiovascular diseases (9, 10). The global prevalence of IR has surged alongside obesity and type 2 diabetes mellitus (T2DM) (11–14). According to the National Health and Nutrition Examination Survey (NHANES), approximately 40% of individuals aged 18–44 years are affected by IR (15), while the American College of Endocrinology reports that over 90% of individuals with T2DM in the United States exhibit IR (16).

Skeletal muscle is the primary organ responsible for insulin-regulated glucose disposal, and greater muscle mass is generally associated with improved insulin sensitivity (17–19). In the context of IR, insulin signaling in skeletal muscle is impaired, resulting in reduced glucose uptake and abnormal protein metabolism, which accelerates muscle mass loss. This depletion of muscle mass further exacerbates insulin resistance, establishing a vicious cycle of deterioration (17). Longitudinal cohort studies state that increased Homeostasis Model Assessment of Insulin Resistance (HOMA-IR) is independently associated with reduced lean body mass and skeletal mass in older men (20). Other indices, such as the relative skeletal muscle index and extremity skeletal mass index, have also been identified as predictive markers for IR risk (21–23). However, current methods for assessing muscle mass, such as CT, MRI, and dual-energy X-ray absorptiometry, are expensive and not easily accessible (24, 25), emphasizing the need for more accessible biomarkers.

Serum creatinine/cystatin C (Cr/CysC) has been proposed as an indicator of sarcopenia, with its validity confirmed through CT imaging in older individuals, cancer patients, and individuals with diabetes (26–28). Unlike traditional methods, such as the HOMA-IR index for assessing IR, Cr/CysC is simple, cost-effective, and requires only serum measurements of Cr and CysC, making it highly applicable across diverse clinical settings.

While several reports have stated an association between Cr/CysC and IR, the majority of studies have focused on populations with specific conditions, such as diabetes, osteoporosis, or metabolic syndrome (29–32). Despite existing evidence supporting Cr/CysC as a muscle mass marker, its direct association with IR and all-cause mortality in general populations remains unclear. This study aims to address this gap by analyzing nationally representative cohort data.

Accumulating evidence suggests that Cr/CysC may be associated with all-cause mortality. As a surrogate marker of muscle mass, low Cr/CysC levels could reflect underlying sarcopenia and poor metabolic reserves, both independently associated with increased mortality risk (33, 34). However, the combined evaluation of Cr/CysC with respect to both IR and mortality in general populations remains underexplored. This study aims to fill that gap by examining the associations between Cr/CysC, IR, and all-cause mortality in a nationally representative U. S. cohort.

The hyperinsulinemic–euglycemic clamp is considered the gold standard for assessing IR. However, this method is invasive, costly, and time-consuming, making it impractical for routine clinical use. In recent years, the triglyceride-to-high-density lipoprotein cholesterol (TG/HDL) ratio has been repeatedly validated as a reliable surrogate marker for IR (35, 36). Studies indicate that a TG/HDL ratio of 3.5 provides optimal predictive value for IR, demonstrating high sensitivity and specificity in identifying insulin-resistant individuals (37). Therefore, this study adopts a TG/HDL ratio ≥ 3.5 as the diagnostic criterion for IR.

## Methods

### Data source and inclusion procedures

Data were sourced from the NHANES, organized by the National Center for Health Statistics, to inspect the nutritional and health conditions of U. S. citizens. The survey employs a rigorous multistage probability sampling to ensure that the data accurately represent diverse demographic groups.

**Figure 1:**
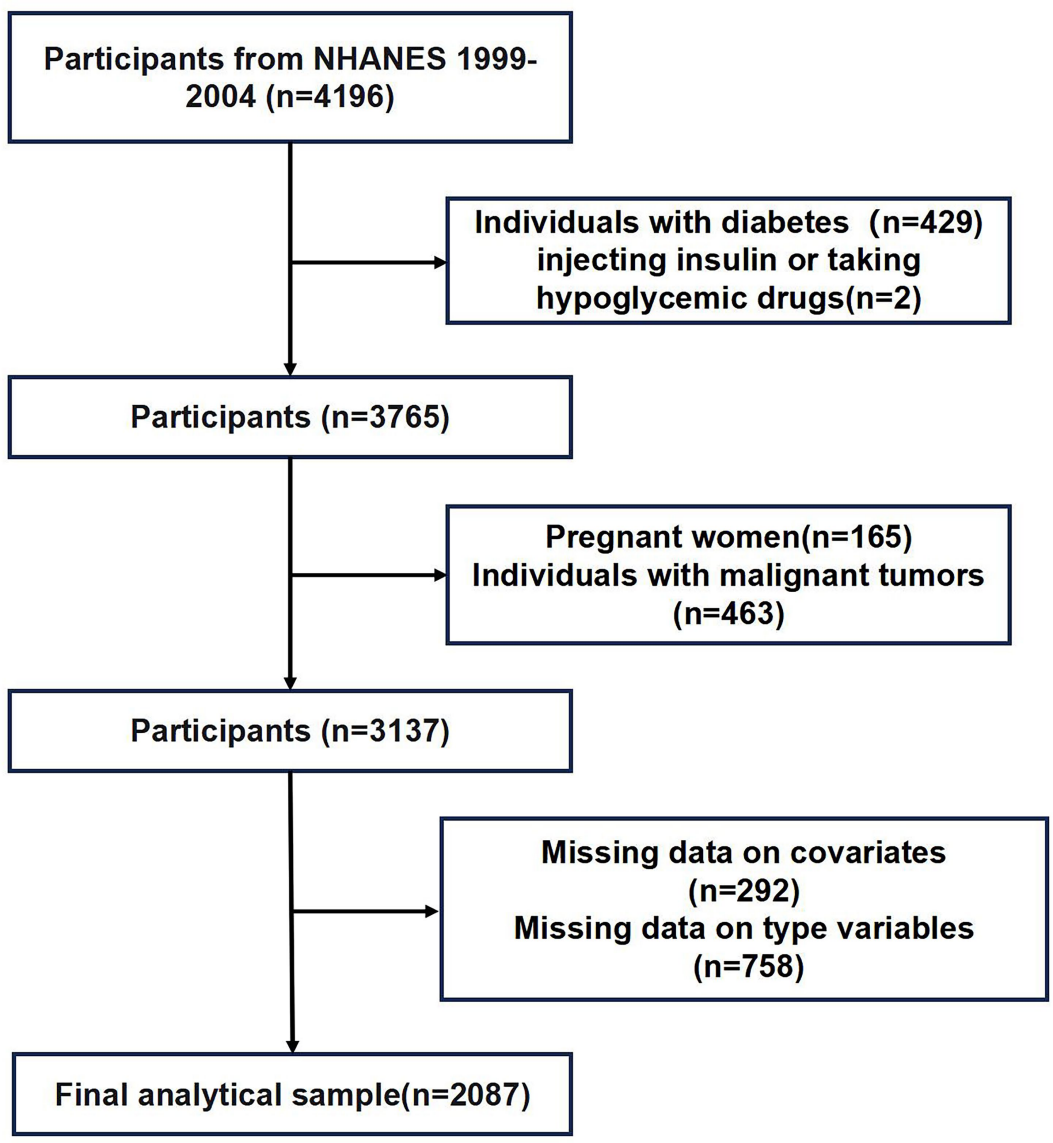
Inclusion procedures in NHANES 1999–2004.

Data from three NHANES cycles (1999–2004) were employed. After initial inclusion of 4,196 participants, pregnant women (*n* = 165), patients with malignant neoplasms (*n* = 463), diabetes mellitus (*n* = 429), insulin or glucose-lowering medications (*n* = 2), missing continuous variables (*n* = 292), and missing categorical variables (*n* = 758) were excluded. Finally, 2,087 participants were left. The inclusion procedures are displayed in Figure 1.

### Measurement of the Cr/CysC ratio

Serum creatinine was determined by the kinetic Jaffe method. Cystatin C in serum was determined by cystatin C immunoassay (Siemens Healthineers Diagnostics) (38).

### Definition of IR

TG/HDL is considered a surrogate index of IR, with a ratio > 3.5 predictive of IR (39).

### Assessment of covariates

Covariates related to IR were also recorded (40–43), encompassing age, sex, ethnicity, poverty income ratio (PIR) (≤3, >3), marriage, education, alcohol use, tobacco use, hypertension (yes, no), body mass index (BMI), waist circumference, insulin, glucose, HDL-C, total cholesterol (TC), low-density lipoprotein cholesterol (LDL-C), TG, Cr, CysC, and Cr/CysC. PIR ≤ 3 was considered low to moderate income, and PIR > 3.0 was considered high income (42). BMI (kg/m^2^) was divided into <30 kg/m^2^ (non-obese) and ≥30 kg/m^2^ (obese individuals) (44).

### Mortality evaluation

To determine all-cause death, we accessed the NHANES mortality dataset, which is linked to the National Death Index. First, causes of death were classified according to ICD-10 codes (45). The follow-up duration was from the participant’s examination at the ambulatory screening center to death or until 31 December 2019, whichever occurred earlier.

### Data synthesis

This study incorporated weights in all analyses to acquire nationally representative estimates for U.S. citizens. Descriptive statistics for categorical estimates were delineated as weighted percentages, and pairwise comparisons were performed using chi-square tests.

The analysis involved three models with stepwise adjustments: the crude model was not adjusted; Model 1 adjusted for sex, age, and ethnicity; Model 2 further adjusted for PIR, marriage, education, drinking, smoking, hypertension, and BMI.

Logistic regression analyses with IR as a dichotomous outcome were employed to characterize the risk estimates in odds ratios (ORs) and 95% confidence intervals (CIs). Additionally, the linear trend across tertiles was tested by introducing tertiles as a single continuous variable into the model. Weighted linear regression models were employed to analyze continuous outcome variables. The Cox proportional hazards model was utilized to estimate hazard ratios (HRs) and 95% CIs between Cr/CysC and all-cause mortality. The dose–response relationship between Cr/CysC and IR was studied using restricted cubic splines (RCS), with the median as the inflection point and five selected nodes. The relationship was tested separately in the overall population and subgroups (men and women). All data analyses were conducted using R version 4.2.2 and SPSS 27, with *p* < 0.05 indicating statistical significance.

## Results

### Baseline characteristics

The sociodemographic characteristics and clinical data are listed in Table 1. Compared to their non-IR counterparts, individuals with IR were more often men, non-Hispanic White or of other ethnicities, and smokers. They also exhibited larger waist circumference, a higher prevalence of metabolic disorders (such as hypertension, high BMI, high insulin uptake, and high plasma glucose), poorer blood lipid profiles (higher TC, TG, LDL-C, and low HDL-C), and higher Cr and CysC levels but lower Cr/CysC ratios.

**Table 1:** Weighted characteristics and measured data from NHANES 1999–2004.

Characteristics	All	No-IR	IR	P*
	(*n* = 2087)	(*n* = 1,509)	(n = 578)	
Age				
20–39	617 (43.1)	470 (43.9)	147 (40.8)	0.309
40–60	620 (42.1)	453 (42.1)	167 (41.9)	
> 60	850 (14.9)	586 (14.0)	264 (17.3)	
Sex				
Male	1,137 (51.2)	760 (46.0)	377 (66.4)	<0.001*
Female	950 (48.8)	749 (54.0)	201 (33.6)	
Ethnicity				
Non-Hispanic White	1,163 (75.8)	844 (74.7)	319 (79.1)	0.001*
Non-Hispanic Black	353 (9.1)	296 (10.6)	57 (4.8)	
Other ethnicities	571 (15.1)	369 (14.7)	202 (16.2)	
Marriage				
Married or living with a partner	1,316 (67.5)	926 (66.2)	390 (71.3)	0.125
Not married nor living with a partner	771 (32.5)	583 (33.8)	188 (28.7)	
PIR				
> 3	896 (54.6)	657 (53.5)	239 (58.0)	0.155
≤ 3	1,191 (45.4)	852 (46.5)	339 (42.0)	
Education				
High school graduate or higher	1,475 (84.6)	1,096 (85.0)	379 (83.5)	0.488
Less than high school	612 (15.4)	413 (15.0)	199 (16.5)	
Drink (Past 12 months)				
0 time	473 (16.9)	321 (16.0)	152 (19.4)	0.177
≤ 12 times	1,561 (80.4)	1,151 (81.5)	410 (77.3)	
> 12 times	53 (2.7)	37 (2.4)	16 (3.4)	
Smoke				
No	915 (45.9)	707 (47.9)	208 (39.8)	0.017*
Yes	1,172 (54.1)	802 (52.1)	370 (60.2)	
Hypertension				
No	1,401 (75.1)	1,045 (78.0)	356 (66.7)	0.001*
Yes	686 (24.9)	464 (22.0)	222 (33.3)	
BMI (kg/m^2^)				
< 30	1,434 (71.6)	1,093 (75.6)	341 (60.0)	<0.001*
≥ 30	653 (28.4)	416 (24.4)	237 (40.0)	
Waist circumference (WC)(cm)	95.80 (15.19)	93.26 (14.88)	103.19 (13.58)	<0.001*
Insulin (uU/mL)	10.49 (9.00)	8.82 (6.55)	15.37 (12.65)	<0.001*
Plasma glucose (mmol/L)	97.12 (17.76)	95.35 (15.50)	102.24 (22.36)	<0.001*
TC (mg/dL)	198.39 (38.12)	195.17 (36.92)	207.73 (39.99)	<0.001*
HDL-C (mg/dL)	52.86 (14.89)	57.21 (14.13)	40.22 (8.42)	<0.001*
LDL-C (mg/dL)	120.19 (33.47)	118.52 (32.73)	125.01 (35.11)	0.003*
TG (mg/dL)	126.79 (66.66)	97.27 (35.90)	212.51 (61.02)	<0.001*
Cr (mg/dL)	0.86 (0.30)	0.84 (0.30)	0.90 (0.31)	0.001*
CysC (mg/L)	0.79 (0.23)	0.77 (0.22)	0.85 (0.25)	<0.001*
Cr/CysC	1.12 (0.35)	1.13 (0.36)	1.09 (0.34)	0.002*

*p < 0.05. Unweighted n, all other analyses are weighted.

### Associations between Cr/CysC and IR

In weighted logistic regression modeling (Table 2), Cr/CysC was greatly and negatively associated with IR risk and remained robust (OR = 0.35, 95% CI: 0.15–0.79) in Model 2. Consistently, IR risk was considerably lower in the highest quartile (OR = 0.48, 95% CI: 0.32–0.73). A linear trend was unveiled for this association (*P f*or trend = 0.001). Cr/CysC ratio may be a protective indicator for assessing IR risk.

**Table 2:** Weighted logistic regression of Cr/CysC and TG/HDL.

Cr/CysC	Crude	Model 1	Model 2
Continuous	0.60 (0.38, 0.96) 0.033*	0.32 (0.15, 0.68) 0.004*	0.35 (0.15, 0.79) 0.013*
Categories			
T1	Ref	Ref	Ref
T2	0.88 (0.64, 1.20) 0.402	0.70 (0.49, 0.98) 0.040*	0.72 (0.51, 1.02) 0.061
T3	0.71 (0.50, 0.99) 0.044*	0.46 (0.30, 0.71) < 0.001*	0.48 (0.32, 0.73) 0.001*
*P for trend*	0.84 (0.71, 0.99) 0.043*	0.68 (0.54, 0.84) < 0.001*	0.69 (0.56, 0.85) 0.001*

Crude Model: Non-adjusted model; Model 1: adjusted for age, sex, and ethnicity; Model 2: adjusted for age, sex, ethnicity, marriage, PIR, education, drinking, smoking, hypertension, and BMI. *P < 0.05.

In weighted linear regression (Table 3), continuous Cr/CysC was negatively associated with TG/HDL (*β* = −0.38, SE = 0.14) in the unadjusted model, especially in the highest quartile (T3: *β* = −0.38, SE = 0.17). The correlation was enhanced after adjusting for demographic factors (age, sex, and ethnicity; continuous Cr/CysC: *β* = −0.67, SE = 0.23; T3: *β* = −0.72, SE = 0.18; *P* for trend, *p <* 0.001). After further adjustment for confounders, the significant association was maintained (continuous: *β* = −0.55, SE = 0.20; T3: *β* = −0.60, SE = 0.17), suggesting a robust negative association between Cr/CysC and TG/HDL.

**Table 3:** Weighted linear regression of Cr/CysC and TG/HDL.

Index	Model	Cr/CysC	
		Beta (SE)	*P*
Crude	Continuous	−0.38 (0.14)	0.009*
	Categories		
	T1	Ref	
	T2	−0.19 (0.14)	0.175
	T3	−0.38 (0.17)	0.031*
	*P for trend*	−0.19 (0.08)	0.032*
Model 1	Continuous	−0.67 (0.23)	0.007*
	Categories		
	T1	Ref	
	T2	−0.36 (0.14)	0.012*
	T3	−0.72 (0.18)	<0.001*
	*P for trend*	−0.36 (0.09)	<0.001*
Model 2	Continuous	−0.55 (0.20)	0.010*
	Categories		
	T1	Ref	
	T2	−0.29 (0.13)	0.031*
	T3	−0.60 (0.17)	0.001*
	*P for trend*	−0.30 (0.08)	0.001*

SE: standard error, *P < 0.05.

**Figure 2:**
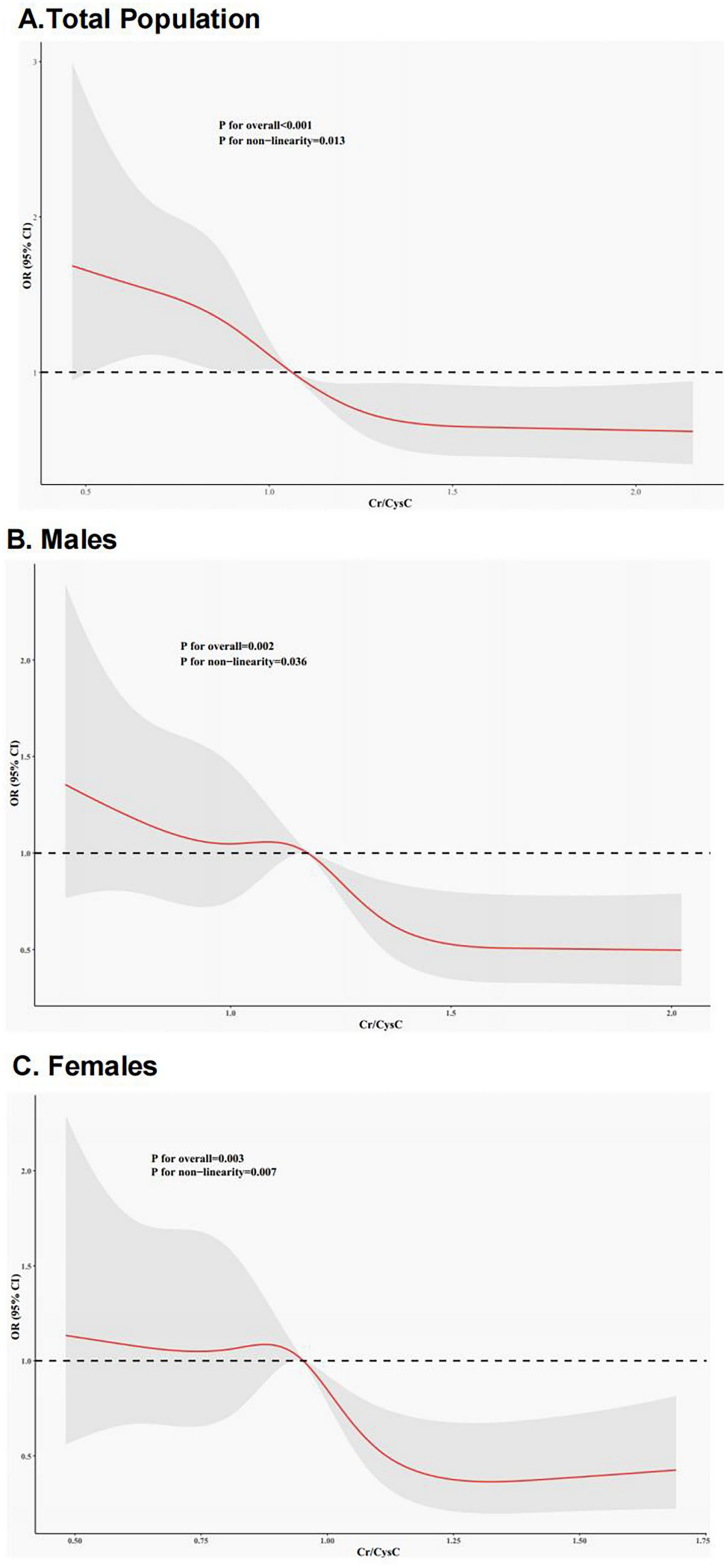
RCS curves for the link between Cr/CysC and IR. **(A)** Entire Cohort; **(B)** Females; **(C)** Males. Red lines represent ORs, and gray areas denote 95%CIs.

RCS modeling proved a non-linear link between Cr/CysC and IR risk (Figure 2). All three plots revealed that Cr/CysC was negatively linked with IR risk, with non-linear associations in men, women, and the whole population. IR risk was greater at lower Cr/CysC. The non-linear trend was particularly pronounced in women and the whole population. The turning points for total populations, men, and women were Cr/CysC = 1.06, 1.18, and 0.95, respectively. These inflection points suggest the differential effects Cr/CysC ratio on the outcomes of different sexes.

### Associations between Cr/CysC and mortality

This study illustrated a linear inverse link of Cr/CysC to all-cause death (Table 4). In continuous variable analysis, each unit increase in Cr/CysC was linked with a 53% decline in death risk (HR = 0.47, 95%CI: 0.30–0.69). Tertile comparisons exhibited progressively lower risks in T2 (0.77, 0.63–0.94) and T3 (0.73, 0.56–0.95) versus T1 (both *p* < 0.05), with a notable linear trend (*P* for trend = 0.007). Similar patterns were unveiled in IR subgroups.

**Table 4:** Cox regression of Cr/CysC and all-cause mortality.

Index	Cr/CysC Variable	Cr/CysC	
		HR (95%CI)	*P*
Total	Continuous	0.47 (0.31–0.69)	<0.001*
	Categories		
	T1	Ref	
	T2	0.77 (0.63–0.94)	0.009*
	T3	0.73 (0.56–0.95)	0.018*
	*P for trend*	0.84 (0.74–0.95)	0.007*
Without IR	Continuous	0.42 (0.26–0.67)	<0.001*
	Categories		
	T1	Ref	
	T2	0.76 (0.61–0.96)	0.019*
	T3	0.70 (0.52–0.96)	0.026*
	*P for trend*	0.82 (0.71–0.96)	0.011*
With IR	Continuous	0.60 (0.30–1.19)	0.145
	Categories		
	T1	Ref	
	T2	0.76 (0.52–1.11)	0.160
	T3	0.84 (0.52–1.37)	0.483
	*P for trend*	0.89 (0.70–1.13)	0.343

*P < 0.05.

Similarly, in the population without IR, the continuous variable analysis revealed a negative link of Cr/CysC levels to all-cause death (HR = 0.42, 95% CI: 0.26–0.67). Death risk in T2 (0.76, 0.61–0.96) and T3 groups (0.70, 0.52–0.96) was significantly lowered. However, in IR populations, the continuous variable analysis did not reveal a pronounced link (0.60, 0.30–1.19). Similarly, categorical variable analysis did not reveal considerable differences in mortality risk between the T2 (0.76, 0.52–1.11) or T3 (0.84, 0.52–1.37) groups and the T1 group.

Overall, the results suggest that the Cr/CysC was negatively linked with all-cause death in total populations and non-IR individuals, but no pronounced association was noticed in IR populations.

### Subgroup analysis

The results manifested a significant inverse association between Cr/CysC and study outcomes (OR = 0.60, 95% CI: 0.43–0.82), indicating that higher Cr/CysC levels were statistically correlated with lower odds of adverse outcomes. In the age stratification, pronounced associations were unveiled only in the 40–60 years group. In the sex stratification, both men and women presented notable negative associations, especially in women. In the ethnicity stratification, other ethnicities showed notable associations. Negative associations of Cr/CysC with outcomes were significant in those who were unmarried or not living with a partner and in those with low PIR. Significant associations were also shown among moderate drinkers and smokers. In addition, the associations were particularly significant among those without hypertension and those with a BMI < 30 (*p* < 0.05) (see Table 5).

**Table 5:** Subgroup analysis of Cr/CysC and IR.

Variables	*N* (%)	Case/control	OR (95%CI)	*P*
All patients	2087 (100.00)	578/1509	0.60 (0.43 ~ 0.82)	0.002*
Age				
20–39	617 (29.56)	147/470	0.72 (0.39 ~ 1.32)	0.289
40–60	620 (29.71)	167/453	0.50 (0.26 ~ 0.95)	0.034*
> 60	850 (40.73)	264/586	0.81 (0.51 ~ 1.29)	0.382
Gender				
Men	1,137 (54.48)	377/760	0.46 (0.29 ~ 0.71)	<0.001*
Women	950 (45.52)	201/749	0.21 (0.11 ~ 0.41)	<0.001*
Ethnicity				
Non-Hispanic White	1,163 (55.73)	319/844	0.92 (0.64 ~ 1.33)	0.659
Non-Hispanic Black	353 (16.91)	57/296	0.84 (0.35 ~ 2.04)	0.699
Other ethnicities	571 (27.36)	202/369	0.40 (0.22 ~ 0.74)	0.003*
Marriage				
Married or living with a partner	1,316 (63.06)	390/926	0.71 (0.49 ~ 1.02)	0.065
Not married nor living with a partner	771 (36.94)	188/583	0.39 (0.22 ~ 0.70)	0.001*
PIR				
> 3	896 (42.93)	239/657	0.80 (0.52 ~ 1.24)	0.323
≤ 3	1,191 (57.07)	339/852	0.47 (0.31 ~ 0.74)	<0.001*
Education				
High school graduate or higher	1,475 (70.68)	379/1096	0.65 (0.44 ~ 0.95)	0.025*
Less than high school	612 (29.32)	199/413	0.60 (0.34 ~ 1.07)	0.083
Drink (Past 12 months)				
0 times	473 (22.66)	152/321	0.83 (0.42 ~ 1.65)	0.595
≤ 12 times	1,561 (74.80)	410/1151	0.45 (0.30 ~ 0.67)	<0.001*
> 12 times	53 (2.54)	16/37	1.38 (0.73 ~ 2.60)	0.324
Smoking status				
No	915 (43.84)	208/707	0.61 (0.37 ~ 0.99)	0.047*
Yes	1,172 (56.16)	370/802	0.65 (0.43 ~ 0.99)	0.044*
Hypertension				
No	1,401 (67.13)	356/1045	0.51 (0.34 ~ 0.77)	0.001*
Yes	686 (32.87)	222/464	0.85 (0.56 ~ 1.27)	0.422
BMI (kg/m^2^)				
< 30	1,434 (68.71)	341/1093	0.64 (0.43 ~ 0.95)	0.026*
≥ 30	653 (31.29)	337/416	0.64 (0.38 ~ 1.08)	0.095

*P < 0.05.

## Discussion

This study explored the associations between Cr/CysC, IR, and all-cause death based on the NHANES databases and uncovered that higher Cr/CysC was significantly and inversely linked with IR, exhibiting a non-linear dose–response link. Cr/CysC was negatively associated with all-cause death in non-IR populations but not in the IR population. Subgroup analyses evinced that an inverse link of Cr/CysC with IR was more notable in the middle-aged, female, and non-hypertensive populations.

This study proved an independent non-linear link between elevated Cr/CysC and low IR risk. Consistently, a CHARLS study of 5,055 middle-aged and older adults reported that enhanced Cr/CysC was related to diminished risk of diabetes and cardiometabolic comorbidities (33). A cohort study of American adults found that low Cr/CysC was related to elevated cardiovascular event risk (34). The potential mechanisms may be that Cr/CysC is an index of muscle mass, reflecting the contribution of skeletal muscle to insulin-regulated glucose uptake, and reduced muscle mass may exacerbate IR through ectopic lipid deposition and inflammation (17, 19). Furthermore, non-linear analysis indicated a threshold effect of Cr/CysC, where values < 1.06 corresponded to rapidly escalating IR risk, emphasizing the need for early intervention in low Cr/CysC populations.

We found that lower Cr/CysC was markedly connected with greater all-cause death, especially in non-IR populations. Consistently, a cohort study involving 1,476 participants found that low Cr/CysC was linked to enhanced mortality risk in patients with T2DM (46). Another cohort study of 12,914 U.S. adults reported negative correlations of Cr/CysC with deaths from all causes, cardiovascular events, and cancer (47). As a surrogate index of skeletal muscle mass, lower Cr/CysC may reflect muscle loss, leading to reduced insulin sensitivity, increased chronic inflammation, and ectopic lipid deposition (17–19), thereby impairing glucose homeostasis and accelerating metabolic disorders (18). Notably, this association disappeared in the IR population, potentially due to IR-related chronic inflammation, abnormal insulin signaling, and renal dysfunction (48–50).

Lower mortality risk with higher Cr/CysC levels aligns with the skeletal muscle’s role in maintaining metabolic health. This inverse association may be mechanistically explained by the critical role of skeletal muscle in regulating glucose metabolism, insulin sensitivity, and overall physiological resilience (1). Low Cr/CysC, as a proxy for reduced muscle mass, could indicate poor metabolic capacity, leading to higher systemic inflammation, impaired insulin signaling, and increased vulnerability to comorbidities (18). Notably, the absence of a significant association in the insulin-resistant subgroup might be attributed to the complex interplay between chronic inflammation, endothelial dysfunction, and renal impairment commonly present in this population, potentially attenuating the predictive value of Cr/CysC for mortality risk (9).

We also acknowledge recent mechanistic studies that further elucidate the complex pathophysiology of IR. For example, selenoproteins have emerged as redox-sensitive modulators involved in lipid and glucose metabolism, with dysregulated expression implicated in oxidative stress and IR progression (51). Moreover, epigenetic regulation may also contribute to IR heterogeneity: a recent study found that LncRNA Kcnq1ot1 levels were significantly elevated in patients with T2DM and correlated with inflammatory cytokines IL-6 and IL-*β*, suggesting a role in metabolic inflammation (52). These molecular insights may complement future biomarker-based risk stratification strategies.

Our findings further underscore the value of incorporating Cr/CysC as a biomarker in broader clinical frameworks. A recent machine-learning model (53) demonstrated strong predictive capacity for diabetic macroangiopathy using routine clinical features. While our study did not focus on complications, the potential utility of Cr/CysC in such ML-based risk prediction tools represents a valuable direction for future research, particularly for the early identification of high-risk individuals.

Additionally, although our study is observational in nature, we recognize that interventions targeting the biological underpinnings of IR are of significant interest. In particular, traditional Chinese medicine formulations such as Liuwei Dihuang Decoction have shown promise in improving IR through activation of the PI3K/Akt signaling pathway in preclinical models (54). These complementary strategies may align with Cr/CysC-based assessments in the future. Similarly, innovative therapies addressing complications of diabetes, such as glucose-responsive photodynamic analgesic gels for treating diabetic abscesses (55), underscore the expanding therapeutic landscape of diabetes care and its intersection with metabolic regulation. Although these interventions fall beyond the scope of our cohort-based analysis, they highlight the interconnected biological systems that influence IR, muscle mass, inflammation, and metabolic outcomes.

This study identified a particularly strong inverse association between Cr/CysC and IR in middle-aged individuals (40–60 years), women, and those without hypertension. The accelerated decline in skeletal muscle mass that typically occurs during middle age may amplify potential metabolic benefits associated with higher Cr/CysC levels in this population (20). Sex differences are likely influenced by the regulatory role of estrogen in muscle protein synthesis (23), with muscle loss in women rendering Cr/CysC fluctuations more sensitive to metabolic disturbances.

Additionally, the stronger association observed in non-hypertensive individuals may be explained by the absence of hypertension-related vascular endothelial dysfunction, which can impair skeletal muscle microcirculatory perfusion and limit insulin-mediated glucose uptake (1, 9). In hypertensive patients, this pathological state may obscure the potential contribution of muscle mass, as reflected by Cr/CysC, to insulin sensitivity. Furthermore, chronic inflammation and oxidative stress commonly present in hypertensive individuals may independently promote IR through mechanisms, such as muscle atrophy and ectopic lipid accumulation (48, 49).

These findings collectively suggest that Cr/CysC, as a surrogate biomarker of muscle mass, may serve as a useful indicator associated with IR risk in individuals without significant vascular pathology. Consequently, Cr/CysC may have potential utility as an auxiliary marker in identifying individuals with increased IR risk in specific high-risk populations, including middle-aged individuals, women, non-hypertensive people, those with lower socioeconomic status, moderate drinkers or smokers, and those with a BMI < 30.

Our study has several notable strengths. First, it utilizes data from the large-scale NHANES database, ensuring strong representativeness and broad applicability. Second, the use of RCS models and subgroup analyses reveals non-linear associations and sex-specific differences, providing support for personalized prevention strategies. Additionally, Cr/CysC, as a low-cost and easily accessible biomarker, shows potential for clinical application in the risk stratification of IR.

However, this study has several limitations. First, its cross-sectional design prevents causal inferences, which should be addressed in future longitudinal or interventional studies. Second, the serum creatinine and cystatin C measurements in NHANES 1999–2004 were not traceable to the IDMS or ERM-DA471/IFCC reference standards. Although standardized and calibrated methods were used, this may introduce measurement bias; however, internal comparisons remain valid. Third, potential confounding from unmeasured factors affecting cystatin C levels, such as certain medications and clinical conditions, could not be fully controlled due to data limitations. Fourth, since the study population was based in the United States, the generalizability of these findings to other ethnicities and regions requires confirmation in large, multicenter, and international cohorts. Additionally, although TG/HDL is a convenient and widely accepted surrogate marker for IR, it can be influenced by lipid metabolism disorders, inflammation, and medication use, with variable diagnostic accuracy across different populations. To validate the robustness of our findings, a sensitivity analysis using the TyG index was performed, which yielded consistent results. Although TG/HDL > 3.5 has been widely used as a surrogate marker for IR, its diagnostic accuracy may vary across racial and ethnic groups due to genetic and metabolic differences in lipid profiles. While this threshold has demonstrated utility in multiethnic cohorts, further validation using subgroup analyses or development of ethnicity-specific cutoffs may enhance its diagnostic precision and generalizability.

Moreover, future research should focus on clarifying the biological mechanisms linking skeletal muscle mass, Cr/CysC, and IR through cellular, animal, and clinical studies. Prospective research is also needed to explore the feasibility and clinical value of applying Cr/CysC as a simple, accessible tool for early IR risk assessment, particularly in resource-limited settings.

## Conclusion

The present study confirmed that Cr/CysC was independently and inversely linked with IR and all-cause mortality and revealed a significant protective effect in specific subgroups. This low-cost indicator has the potential for IR risk stratification, especially in resource-limited areas.

## Data Availability

The original contributions presented in the study are included in the article/Supplementary material, further inquiries can be directed to the corresponding author.
