# Supplementary material for: Association of serum creatinine/cystatin C ratio with insulin resistance and all-cause mortality: a national cohort analysis
Source: Front Nutr. 2025 Aug 29;12:1619618. doi: 10.3389/fnut.2025.1619618 (PMC12426255; doi:10.3389/fnut.2025.1619618)
Supplement: Supplementary file 1 [file Table_1.DOCX]

| Multivariate Analyses for association between with Creatinine/Cystatin C Ratio and TyG | | | |
| --- | --- | --- | --- |
| Index |  | Cr/CysC | |
|  |  | OR (95%CI) | P value |
| Crude Model | Continuous | 0.58 (0.35, 0.97) | 0.038* |
|  | Categories |  |  |
|  | T1 | ref |  |
|  | T2 | 0.85 (0.60, 1.20) | 0.342 |
|  | T3 | 0.62 (0.42, 0.91) | 0.015* |
|  | *P for trend* | 0.78 (0.65, 0.95) | 0.012* |
| Model 1 | Continuous | 0.56 (0.31, 1.03) | 0.063 |
|  | Categories |  |  |
|  | T1 | ref |  |
|  | T2 | 0.82 (0.55, 1.22) | 0.319 |
|  | T3 | 0.57 (0.35, 0.95) | 0.031* |
|  | *P for trend* | 0.75 (0.59, 0.97) | 0.028* |
| Model 2 | Continuous | 0.64 (0.42, 1.00) | 0.050 |
|  | Categories |  |  |
|  | T1 | ref |  |
|  | T2 | 0.89 (0.60, 1.32) | 0.558 |
|  | T3 | 0.65 (0.41, 1.02) | 0.060 |
|  | *P for trend* | 0.80 (0.64, 1.00) | 0.053 |

* *P*<0.05.
